# Exploring the landing speed of digital society PPP projects: A continuous-time event history analysis of 300 projects

**DOI:** 10.1371/journal.pone.0329180

**Published:** 2025-09-02

**Authors:** Jun Yang, Xiaomei Hu, Zhanyu Liu

**Affiliations:** 1 School of Finance, Hunan University of Finance and Economics, Changsha, China; 2 School of International Relations and Public Affairs, Fudan University, Shanghai, China; The Chinese University of Hong Kong, HONG KONG

## Abstract

The Public-Private Partnership (PPP) model has become a viable alternative or supplement to traditional approaches in the development of a digital society. However, PPP projects in this domain often face significant landing (the launch of project implementation, typically marked by contract signing and the commencement of operational activities) challenges. Understanding the factors that influence the speed of project landing is thus of considerable practical importance. Based on the theoretical framework of Van Meter and Van Horn, we propose a causal mechanism linking the policy implementation system to the landing speed of digital society PPP projects. Using data from 300 projects, the study empirically tests this mechanism through continuous-time event history analysis. The results indicate that, compared with organizational and actor factors, material resources such as government fiscal resources and regional financial resources have a relatively weaker impact on landing speed. In contrast, internal goal consensus on innovation, internal government coordination, government-business relationship quality, leadership performance demand, and corporate social responsibility all significantly promote faster landing. Conversely, due to low project profitability and market barriers to social capital participation, information enterprise development is negatively associated with landing speed. We provide targeted policy recommendations to enhance the efficiency and timeliness of PPP-driven digital society initiatives.

## 1. Introduction

The digital society represents a new social paradigm that has emerged following the transitions from agrarian, industrial, and information societies. Its development encompasses a range of initiatives, including smart and accessible digital public services, smart cities, and digital villages [1]. Both theoretical discourse and practical experience indicate that the Public–Private Partnership (PPP) model has become a viable alternative or complement to traditional approaches in many areas of digital society construction. Specifically, the involvement of private capital not only enhances the financial capacity of development projects but also effectively compensates for governmental shortcomings in infrastructure, human resources, and technical expertise. In this context, the practical necessity of adopting the PPP model for digital society development has been increasingly recognized by local governments across China. As of now, 28 provincial-level administrative regions in China have implemented the PPP model in digital society-related projects. However, it is worth noting that PPP projects in this domain frequently encounter difficulties in landing, including delayed or failed execution. In this study, “landing” refers to the launch of project implementation, typically marked by contract signing and the commencement of operational activities. According to publicly available data from the Ministry of Finance’s PPP database, over 50% of digital society PPP projects have yet to achieve landing, with nearly 30% initiated prior to 2017. From this perspective, identifying the key factors that influence the landing speed of these projects holds substantial practical significance for advancing digital transformation.

The concept of a policy implementation system provides a fresh analytical lens to understand the pace at which digital society PPP projects achieve landing. Policy implementation is a critical stage in the policy process—it involves the use of various tools and the integration of diverse resources to achieve policy objectives [2]. In the Chinese context, PPP projects are typically initiated by the government and aimed at delivering public goods, coordinated and managed through administrative directives issued by national or local authorities. From a broad policy perspective, the landing speed of a PPP project essentially reflects the effectiveness of policy implementation. Existing research suggests that administrative actions—including policy implementation—constitute a systemic form of behavior. Only by adopting a holistic, systems-theoretic perspective and analyzing policy implementation as a network of interrelated elements can we accurately grasp the dynamic mechanisms that shape landing performance [3]. In line with this view, scholars both domestically and internationally have explicitly proposed the concept of “policy implementation system” and identified its constituent elements, such as policy objectives, actor characteristics, inter-actor relationships, and the external environment [4]. Therefore, the structural characteristics of the policy implementation system are theoretically significant in influencing the landing speed of PPP projects, particularly those related to digital society development.

Although prior studies have explored the factors influencing PPP project landing—addressing aspects such as government characteristics, the political-economic environment, and government-business relations [5–7]—three key limitations remain. First, from an analytical standpoint, most research lacks a comprehensive systems-theoretic approach, making it difficult to reflect the dynamic interactions between internal and external factors. Second, the literature primarily focuses on generic PPP projects, without considering the unique goals and construction processes of digital society initiatives. Third, much of the research emphasizes annual landing rates, a metric that does not adequately capture the actual speed of project execution [8]. Given these gaps, examining how the policy implementation system affects the speed of digital society PPP project execution provides a valuable contribution to existing PPP literature. We therefore aim to address two central questions: What are the key components of the policy implementation system that influence the execution speed of digital society PPP projects? And through what causal mechanisms do these components exert their influence?

To answer these questions, we employ the policy implementation system model developed by Van Meter and Van Horn to identify the factors shaping PPP project execution speed in the context of digital society development. It proposes a causal framework linking the policy implementation system’s structure to project speed, and empirically tests this framework using continuous-time event history analysis. Data are drawn from digital society-related PPP projects listed on China’s National PPP Information Platform, maintained by the Ministry of Finance. The remainder of this paper is structured as follows: Section 2 outlines the theoretical framework and presents the research hypotheses. Section 3 describes the empirical methodology. Section 4 reports the research findings, and the final section summarizes the conclusions and offers policy recommendations.

## 2. Theory and research hypothesis

### 2.1. Theoretical analysis

Since the mid-1970s, policy science researchers have increasingly turned their attention to the micro-level processes of policy implementation, developing a variety of theoretical models to explain differences in implementation performance. These include process models, interaction models, cyclical models, systems models, and integrated models. Among them, Van Meter and Van Horn are representative figures of the systems model. Drawing on studies of organizational control, judicial decisions, and inter-organizational relationships, they proposed a policy implementation system model. Van Meter and Van Horn identified six key factors that influence implementation performance following policy enactment: *standards and objectives*—that is, the clarity and consensus of policy objectives; *policy resources*; *characteristics of the implementing agencies*; *interorganizational communication and enforcement activities*; *economic, social, and political conditions*; and *the disposition of implementors*—that is, their cognition, reactions, and responsiveness [4].Compared to the widely discussed policy delivery system models of that time, the policy implementation system model offered a more comprehensive framework for explaining the linkage between policy content and implementation outcomes.

In China’s PPP development model, the relationship between providers and users is characterized by a top-down vertical authority structure [9]. As a classic example of a top-down policy implementation approach, Van Meter and Van Horn’s model aligns well with the study of PPP project landing in the context of digital society development. Generally speaking, the external environment—comprising economic, social, and political conditions—tends to remain relatively stable over time and is therefore unlikely to serve as a practical lever for accelerating the landing of digital society PPP projects. As such, we exclude this fifth dimension and concentrate on the dynamic, agent-centered components of the policy implementation system model: internal goal consensus, project resources, actor characteristics, inter-organizational relationships, and actor dispositions. External factors like the economic, social, and political context are instead incorporated as control variables in the econometric analysis.

### 2.2. Research hypothesis

Van Meter and Van Horn argued that during the policy formulation stage, policymakers define implementation standards, procedures, and instruments. If internal actors within the system diverge in their goals, it may hinder the allocation of resources and the development of strategies essential for successful implementation [4]. Digital society PPP projects, which are core to advancing China’s digital transformation strategy, reflect policy goals such as technology-driven development and innovation-led growth [10]. However, government agencies at various levels are often tasked with numerous administrative responsibilities across sectors, and conflicting priorities may compete for attention and resources. Executing agencies may seek to secure additional resources for their own performance or incentives, thereby crowding out support for innovation-oriented goals [11]. Therefore, broad internal support within the government for the innovation-driven development agenda becomes a key endogenous force for securing the necessary resources for digital society PPP projects. Accordingly, the following hypothesis is proposed:

#### Hypothesis 1.

Internal goal consensus on innovation is positively associated with the landing speed of digital society PPP projects.

Government fiscal and financial resources are crucial for the successful implementation of PPP projects. In models where the government pays directly or provides viability gap funding, local governments often bear part of the repayment responsibility [5], making their fiscal capacity a major determinant of risk and return for private partners. Since 2015, China’s Ministry of Finance has stipulated that fiscal expenditures on PPP projects must not exceed 10% of the general public budget, placing significant financial constraints on local governments. Under these conditions, greater fiscal capacity can enhance a project’s ability to control financial risks, thereby improving its appeal to private capital and facilitating project landing. Similarly, a region with more abundant financial resources is more likely to gain support from financial institutions. Their involvement not only helps mitigate project risks but also enhances the predictability of government behavior, making PPPs more attractive to private investors [12], Based on this reasoning, the following hypotheses are proposed:

#### Hypothesis 2.

Government fiscal resources are positively associated with the landing speed of digital society PPP projects.

#### Hypothesis 3.

Regional financial resources are positively associated with the landing speed of digital society PPP projects.

In PPP arrangements, the government serves both as the contracting authority and regulatory entity. Therefore, internal government coordination plays a vital role in project landing. Effective internal coordination fosters an efficient bureaucracy [13], which reduces communication costs, mitigates risks, and attracts private sector participation [14]. For digital society PPP projects, the impact of internal coordination can be summarized in three pathways. During the contract drafting stage, internal coordination allows for better information sharing, enabling the formulation of more comprehensive and scientifically grounded contracts that attract private partners. In the contract approval stage, a well-coordinated government can streamline administrative, legal, and financial procedures, thereby shortening approval timelines and reducing time-related costs. Governments tend to prefer working with private firms that possess strong technical, operational, and managerial capabilities [15]. The average development level of private firms in the market affects the pool of high-quality partners available. Project risks partly stem from agency issues, including suboptimal partner selection due to bounded rationality [16]. Higher-quality firms reduce search and evaluation time for governments. Hence, the following hypotheses are proposed:

#### Hypothesis 4.

Internal government coordination level is positively associated with the landing speed of digital society PPP projects.

#### Hypothesis 5.

Information enterprise development is positively associated with the landing speed of digital society PPP projects.

A sound government–business relationship is essential for the success of digital society PPP projects. Due to the long-term nature of PPP contracts, two key challenges arise. First, failure of a long-term contract imposes not only direct losses on firms but also significant opportunity costs, making trust and communication with the government crucial for participation [17]. Second, the longer the contract duration, the greater the likelihood of major, often unanticipated changes—many of which are not explicitly addressed in the initial contract and require a foundation of mutual trust and relationship maintenance [18]. In projects like smart city development, which have long landing cycles, repeated negotiations and collaboration between the public and private sectors are necessary. Unlike standard commercial arrangements, PPPs are characterized by the dual role of government as both regulator and contracting party, while private firms often occupy a relatively weaker position [19]. As a result, trust becomes essential—only when firms trust the government to honor its commitments will they be motivated to participate. Additionally, digital society PPP projects rely heavily on data, over 80% of which in China is held by government agencies. Effective project execution requires data sharing between the public and private sectors, which in turn depends on a high degree of mutual trust [20]. Therefore, government–business relations significantly affect a project’s ability to attract private capital. Based on this, we propose:

#### Hypothesis 6.

Government-business relationship quality is positively associated with the landing speed of digital society PPP projects.

Finally, local leaders’ pursuit of political achievements and corporate social responsibility (CSR) are two additional dynamic forces that influence project landing. Since 2012, “innovation” has been a national development priority in China. In this context, leveraging technological innovation for social development has become a key performance indicator for evaluating government officials. Research shows that stakeholders with vested interests in PPP project outcomes actively drive project advancement by employing strategic approaches—such as framing projects in favorable terms, legitimizing their necessity, and developing theoretical justifications—to build consensus and align diverse stakeholder expectations [21,22]. As digital society PPP projects embody the notion of innovation-led growth, the political performance demands of local leaders are expected to accelerate project execution. Regarding corporate social responsibility, recent empirical research on public-private partnerships demonstrates that enterprises may engage in collaborative arrangements with governments to deliver public services as part of their social responsibility commitments. This phenomenon is exemplified by the COVID-19 pandemic response, where Shaanxi Weinan Green Agriculture Technology Company partnered with local authorities to implement the “Flying Wing Action” initiative, utilizing unmanned aerial vehicles for disinfection operations. Qualitative interviews with company representatives revealed that their participation was motivated by a desire to “contribute to epidemic prevention efforts” [23], illustrating how CSR considerations can drive private sector engagement in public service delivery. Accordingly, the following hypotheses are proposed:

#### Hypothesis 7.

Leadership performance demand is positively associated with the landing speed of digital society PPP projects.

#### Hypothesis 8.

Corporate social responsibility of local firms is positively associated with the landing speed of digital society PPP projects.

## 3. Empirical Strategies

### 3.1 Event history analysis

To examine the impact of policy implementation systems on the speed at which Public-Private Partnership (PPP) projects in digital society development are launched, we adopt a continuous-time event history analysis (also known as survival analysis). This method focuses on the hazard probability h(t),which is the probability that a subject who has survived up to time t experiences a specific event at time that a subject, having survived until time *t*, experiences a specific event at time  t. This hazard function can be expressed as the ratio of the failure density function f(t) to the survival function S(t) , as shown in Equation (1). The survival function S(t) represents the probability that a case survives beyond time  t, while the failure density f(t) reflects the instantaneous risk of failure at time t . Based on this relationship, a higher failure density and shorter survival time indicate a higher hazard probability. Unlike discrete-time event history analysis, the continuous-time method captures not only the likelihood of an event occurring but also the timing of its occurrence. In the context our study, this method allows for an investigation into both (1) how the characteristics of the policy implementation system influence the likelihood of project landing, and (2) how these characteristics affect the speed at which digital society PPP projects are launched.


$$h(t)=\frac{f(t)}{S(t)}$$ (1)

In selecting observational units, we first employed web-scraping techniques to extract project data from the Project Management Library and the Project Reserve Library of the China PPP Integrated Information Platform. Through this method, a total of 14,100 projects were collected, each with information on 12 key attributes, including project name, location, sector, approval date, development stage, investment size, return mechanism, project overview, scope of cooperation, cooperation duration, operational model, and procurement method. Based on this dataset, the research team manually screened and identified 319 projects relevant to four key domains: digital public services, smart cities, digital rural initiatives, and digital literacy enhancement programs. After further evaluating the completeness of project data and the availability of independent variables, 300 projects were ultimately included in the study. In accordance with the principles of survival analysis, right-censored data were applied to form an unbalanced panel dataset. This process yielded a total of 736 usable observations. Among these, 141 projects had reached landing (i.e., “landed” projects), with provincial-level projects accounting for 21.30%, municipal-level projects 73.15%, and county-level projects 61.11%.

### 3.2 Baseline regression model

To test the hypotheses proposed, the Cox proportional hazards model—a standard method in continuous-time event history analysis—is employed as the baseline regression model. As a semi-parametric model, the Cox model offers several advantages over traditional regression techniques, including not requiring a pre-specified baseline hazard distribution and providing robust coefficient estimates, making it well-suited for this analysis. The baseline regression model is specified as follows:


$$h(t)=h_{0}(t)\;exp\;(\beta_{1-10}{system}_{t-1}+\beta_{x}{controls}_{t-1}$$ (2)

In this equation, h(t) represents the hazard rate, or the probability that a project is implemented at time *t*. The baseline hazard function $h_{0}(t$ depends only on time. The terms ${system}_{t-1}$ and ${control}_{t-1}$ denote the lagged explanatory and control variables, respectively.

### 3.2. Variables and data

#### 3.2.1. Dependent variables.

The dependent variable is the landing speed of digital society PPP projects. To measure this, the duration of each project is calculated as the number of days between the initiation date and the contract signing date, or the censoring date if the contract was not signed. Following Tan et al. (2019), contract signing is considered a valid indicator of project landing [5].

#### 3.2.2. Independent variables.

Based on the theoretical framework proposed by Van Meter and Van Horn, five key dimensions are used to characterize the policy implementation system: internal goal consensus, project resources, actor characteristics, inter-organizational relationships, and actor dispositions.

(1) **Internal goal consensus**. This study uses internal goal consensus on innovation to examine the impact of internal goal consensus. As mentioned above, digital society PPP projects essentially reflect the government’s objectives of “technology-enabled development” and “innovation-driven development.” Therefore, this paper focuses primarily on the goal preferences of regional governments regarding technological innovation in areas where digital society PPP projects are implemented. Since government goal preferences are, to some extent, reflected through resource allocation, this study follows the approach of Li et al. (2018) and measures government internal goal consensus on innovation primarily through the ratio of science and technology fiscal expenditure to general public service expenditure [24].
(2) **Project resources**. Project resources include government fiscal resources and regional financial resources. Government fiscal resources is measured by the natural logarithm of local public fiscal revenue. Regional financial resources, based on Lei et al. (2023), are measured by the ratio of year-end bank loans and deposits to GDP [25].
(3) **Actor characteristics**. This paper explores the impact of implementing actors’ characteristics on the landing of Public-Private Partnership (PPP) projects in the construction of a digital society, focusing on two key dimensions: the internal government coordination level and information enterprise development. Internal government coordination level is a latent variable that cannot be directly observed, but it can be indirectly measured through performance in specific collective actions [26]. One such indicator is the administrative approval center, which reflects departmental coordination and functional integration. Accordingly, the internal government coordination is measured by the number of departments housed within the administrative approval center of the project’s host city. Information enterprise development is assessed by the proportion of information technology professionals relative to the total population, providing an indication of the maturity of the local digital industry.
(4) **Inter-organizational relationships**. We examine the influence of organizational relationships by evaluating government-business relationship quality. The Marketization Index developed by Fan Gang adopts entirely objective indicators to assess the depth and breadth of market-oriented reforms across different regions, offering a reliable reflection of regional progress in marketization [27]. Data prior to 2016 are based on official statistics, while the index for 2017–2019 is estimated following the methodology of Ma Lianfu and colleagues. We use the sub-index “Relationship between Government and Market” as a proxy to evaluate the quality of government-business relationship in various cities, as it effectively captures the nature of their institutional interactions.
(5) **Actor dispositions**. The orientation of actors is assessed through two lenses: leadership performance demand and corporate social responsibility of local firms. Theoretically, as the tenure of an official increases, their capacity to integrate resources and accumulate political capital grows, enhancing their competitiveness and incentivizing them to engage more actively in promotion opportunities [28]. Thus, a longer tenure can serve as an indirect indicator of an leadership’s performance demand. To simplify tenure into integer values—following existing research conventions—if an official assumes office before June 30 of a given year, that year is counted as their first year of tenure (“1”). If they take office after July 1, their influence in that year is minimal, and the tenure is recorded as “0”, with subsequent years added incrementally [29]. Corporate social responsibility of local firms is measured using the average CSR index scores of listed companies located in the project region. These scores are drawn from Hexun’s Social Responsibility Reports for Listed Companies, China’s first professional evaluation platform for CSR. The reports evaluate five dimensions: responsibilities to shareholders, employees, suppliers, customers and consumers, environmental responsibility, and broader social responsibility.

#### 3.2.3. Control variables.

We control for the influence of the project environment on the landing speed of PPP projects from two dimensions: the market technological environment and the external knowledge environment. The market technological environment is measured by the natural logarithm of the number of invention patents granted to high-tech enterprises, while the external knowledge environment is proxied by the number of universities in the region. In addition, drawing on existing studies on PPP project landing, this paper also includes project characteristics as control variables—specifically, project level, demonstration status, investment size, return mechanism, and cooperation duration. Year fixed effects based on the project approval year are also controlled for. Detailed variable definitions are provided in Table 1.

**Table 1:** Variable measurements.

Dimension	Variable Name	Measurement
Dependent variable	Project duration	Number of days elapsed from approval date to observation time (days)
	Project landed	A project is considered on the ground when a project contract is signed
Internal goal consensus	Internal goal consensus on innovation	Financial expenditure on science and technology as a share of general public service expenditure (%)
Project resources	Government fiscal resources	Natural logarithm of government revenue from public finance
	Regional financial resources	Year-end deposit and loan balances of financial institutions as a share of GDP (%)
Actor characteristics	Internal government coordination	Number of departments in the administrative approval center of the city where the project is located (provincial projects take the average value of cities within the province) (pcs)
	Information enterprise development	Share of IT employees in population (%)
Inter-organizational relationships	Government-business relationship quality	Score of “government-market relationship” dimension of marketization index
Actor dispositions	Leadership performance demand	Term of office of local chief executives (in years)
	Corporate social responsibility	Average value of total social responsibility index of listed companies in the region
Control variables	Market technology	Natural logarithm of the number of patents for inventions in high-tech firms
	External knowledge	Number of regional colleges and universities (number)
	Project level	Below municipal level is 1, municipal level is 2, provincial level is 3
	Demonstration status	1 for municipal level, 2 for provincial level, 3 for national level
	Investment size	Total project investment (billion yuan)
	Return mechanism	3 for government payment, 2 for gap subsidy, 1 for others
	Cooperation duration	1 for more than 15 years, 0 for 15 years and below
	Approval year	Fixed effect of year of approval

## 4. Empirical results and discussion

### 4.1. Baseline results

Table 2 presents the descriptive statistics and multicollinearity diagnostics for the main variables. As shown, all variables have variance inflation factors (VIFs) below 10, indicating that multicollinearity is not a serious concern. The descriptive statistics on project duration reveal that among all digital society PPP projects, the maximum duration for projects that remained unimplemented reached 1,856 days. In contrast, the longest duration for implemented projects was 896 days, with an average duration of 369.37 days. These figures highlight the existence of challenges in project landing, including significant delays and difficulties in achieving completion.

**Table 2:** Descriptive statistics and diagnosis of multicollinearity.

Variables	Obs	Mean	Std. Dev.	Min	Max	VIF
Project duration	736	504.29	394.39	2	1856	--
Internal goal consensus on innovation	736	22.83	15.24	7.01	95.89	4.07
Government fiscal resources	736	7.89	0.61	5.47	9.4	6.67
Regional financial resources	736	134.82	30.53	80.3	253.82	2.44
Internal government coordination	736	40.7	18.22	0	95	1.19
Information enterprise development	736	0.25	0.49	0.08	3.9	2.39
Government-business relationship quality	736	5.52	1.62	1.45	8.83	4.45
Leadership performance demand	736	3.01	1.61	1	10	1.12
Corporate social responsibility	736	21.2	3.34	11.71	33.61	1.13
Market technology	736	8.12	1.51	3.74	12.24	7
External knowledge	736	100.99	35.14	12	167	9.93
Project level	736	1.03	0.23	1	3	1.1
Demonstration status	736	0.32	0.88	0	3	1.11
Investment size	736	7.61	11.8	0.07	122.6	1.11
Return mechanism	736	2.07	0.67	1	3	1.25
Cooperation duration	736	0.4	0.49	0	1	1.24

**Fig 1:**
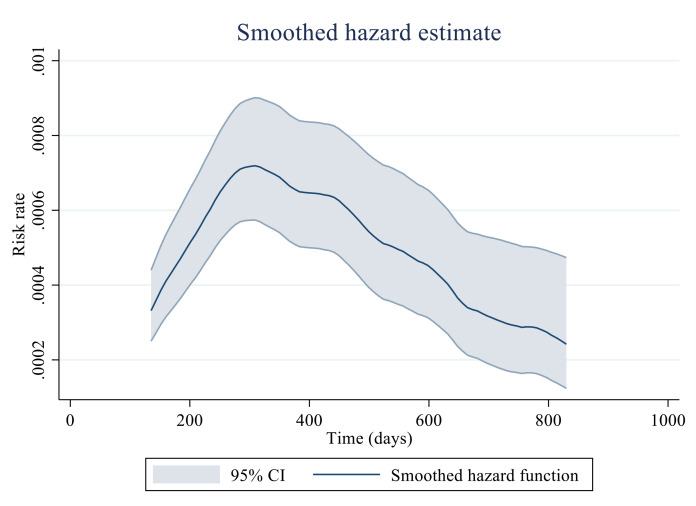
Estimated probability of project landing risk.

To illustrate the distribution of project landing risk over time, we employ a weighted kernel density estimate to construct a smoothed hazard function, as shown in Fig 1. The hazard rate represents the instantaneous probability that a surviving project will be implemented in the next infinitesimally small-time interval. The curve in Fig 1 exhibits an inverted U-shape, with the peak occurring around 300 days, indicating that the probability of project landing is highest at approximately that time. After this point, the likelihood of landing declines, suggesting increasing difficulty as time progresses.

### 4.2. Hypothesis testing

Model 1 in Table 3 reports the results of the baseline regression. Regarding internal goal consensus, the coefficient for internal goal consensus on innovation is significantly positive at the 1% level. A 1% increase in goal alignment leads to a 4% increase in the probability of project landing. This finding supports Hypothesis 1, indicating that higher consistency in innovation goals among stakeholders accelerates project landing.

**Table 3:** Results of hypothesis testing.

Independent Variable	Model 1		Model 2	
	Baseline model		Parametric survival model	
	Coeff. (S.E.)	H.R.	Coeff. (S.E.)	H.R.
Internal goal consensus on innovation	0.039^***^(0.010)	1.040	0.040^***^(0.010)	1.041
Government fiscal resources	0.327(0.359)	1.387	0.246(0.365)	1.279
Regional financial resources	0.008(0.005)	1.008	0.006(0.005)	1.006
Internal government coordination	0.017^***^(0.005)	1.017	0.018^***^(0.005)	1.019
Information enterprise development	−0.861^**^(0.346)	0.423	−0.759^**^(0.336)	0.468
Government-business relationship quality	0.255^**^(0.107)	1.290	0.271^**^(0.107)	1.311
Leadership performance demand	0.094^*^(0.052)	1.098	0.088^*^(0.052)	1.092
Corporate social responsibility	0.068^**^(0.029)	1.070	0.092^***^(0.028)	1.097
Market technology	−0.833^***^(0.159)	0.435	−0.930^***^(0.161)	0.394
External knowledge	0.015^*^(0.008)	1.015	0.019^**^(0.008)	1.019
Project level	−0.138(0.462)	0.871	−0.129(0.469)	0.879
Demonstration status	0.413^***^(0.087)	1.512	0.470^***^(0.088)	1.600
Investment size	0.012^***^(0.005)	1.012	0.014^***^(0.005)	1.014
Return mechanism	0.677^***^(0.158)	1.968	0.643^***^(0.159)	1.902
Cooperation duration	−0.369^*^(0.208)	0.692	−0.435^**^(0.211)	0.647
Year FE	Yes		Yes	
*Observations*	736		736	
pseudo *R*^2^	0.080		0.282	

Notes: Robust standard errors are reported in parentheses; ^*,^^**,^^***^ denote significance levels of 10%, 5% and 1%. All models pass the PH risk assumption.

As for project resources, while the coefficients for government fiscal resources and regional financial resources show the expected direction, their statistical significance does not reach conventional levels. This suggests that in the context of digital society PPP projects, the availability of resources does not significantly influence landing speed, thus failing to support Hypotheses 2 and 3.

In terms of actor characteristics, the level of internal government coordination is positively significant at the 1% level. Specifically, each additional department present in a city’s administrative approval center increases the probability of project landing by 1.7%. This confirms Hypothesis 4, indicating that improved internal government coordination facilitates faster project landing. Unexpectedly, the level of information enterprise development is significantly and negatively associated with project landing at the 5% level. A 1% increase in the information enterprise development is associated with a 57.7% decrease in landing probability, contradicting Hypothesis 5. This suggests that more developed information enterprises may paradoxically hinder the landing of digital society PPP projects.

In the dimension of inter-organizational relationships, the coefficient for government-business relationship quality is significantly positive at the 5% level. Specifically, for each one-point increase in the “government-business relations” score within the marketization index, the probability of a digital society PPP project being implemented increases by 29%. This suggests that stronger and more favorable government-business relations are more likely to facilitate the successful landing of digital society PPP projects— a finding that is broadly consistent with Hypothesis 6.

In terms of actor dispositions, the coefficient for leadership performance demand is significantly positive at the 10% level. That is, with each additional year of a local administrative leader’s tenure, the likelihood of a digital society PPP project being landed in that region increases by 9.8%, a result generally aligned with Hypothesis 7. This implies that the longer a local official remains in office, the stronger the performance-based promotion incentive becomes, thereby increasing the likelihood of promoting project landing. With regard to corporate social responsibility, the coefficient is significantly positive at the 5% level. For each one-point increase in the average CSR index of listed companies within a region, the probability of implementing a digital society PPP project rises by 7.0%. This indicates that stronger corporate social responsibility enhances the likelihood of collaboration with the government in providing public services, thereby increasing the probability of project landing. We conducted Schoenfeld residuals tests and found that several variables – particularly external knowledge (χ² = 8.27, p = 0.004) – violated the proportional hazards assumption, which could bias the estimation results [5]. To address this violation while maintaining modeling precision, we replaced the Cox model with a Weibull parametric survival model. This alternative approach provides three key advantages: (1) it does not require proportional hazards assumptions; (2) enables direct estimation of duration dependence through the shape parameter; and (3) permits straightforward prediction of survival probabilities. The results are presented in Model 2 of Table 3. Importantly, despite this methodological adjustment, our core findings regarding project investment effects remained statistically significant and directionally consistent, demonstrating result robustness.

Hypotheses 2, 3, and 5 were not supported by empirical evidence. Drawing upon existing research, we offer the following explanations. First, in terms of project resources, their role is primarily foundational, and we may have overestimated their impact in the hypotheses. Systems theory suggests that for any given system, the quantity of resource input determines the potential for performance output, while the composition and relationships among actors in the system are key efficiency factors that determine whether that potential can be realized [30]. Therefore, in the context of digital society PPP project landing, inputs such as fiscal and financial resources determine only the potential speed of landing, whereas other organizational conditions are the key variables that drive actual progress. This explains their lack of statistical significance in the hypothesis tests. Second, while the development of information enterprises can objectively facilitate project landing, our hypothesis overlooked the subjective factor of firms’ willingness to participate. Specifically, the development of information enterprises is associated with their competitiveness and investment opportunities in the market, both of which influence firms’ expected returns [31]. However, previous research has pointed out that the current PPP model still suffers from high entry barriers and low returns [32]. Consequently, when expected market returns are high, firms may be less inclined to engage in PPP projects. As a result, the development of information enterprises may be negatively correlated with the speed of digital society PPP project landing. This perspective may provide an alternative explanation for the negative relationship between market development and project landing rates observed in previous PPP studies [33].

### 4.3. Robustness checks

To further enhance the credibility of our findings, additional tests are conducted to examine the potential adverse effects of information enterprise development. To rule out the alternative explanation that the observed negative correlation between information enterprise development and project landing is due to inappropriate measurement indicators, we re-evaluate the relationship using the Information Economy Index, a sub-index from the China Information Society Development Index published by the National Information Center. Furthermore, to verify that the negative relationship between information enterprise development and the speed of project landing is driven by market mechanisms, we substitute information enterprise development with a more general indicator of factor market development and conduct an additional robustness test.

Model 3 in Table 4 presents the results using the alternative measure for information enterprise development. The coefficient remains significantly negative at the 5% level, suggesting that the negative correlation is not the result of measurement bias. Model 4 reports the results using factor market development instead. The coefficient is significantly negative at the 1% level, indicating that the negative relationship between information enterprise development and project landing speed is indeed driven by market dynamics. These findings confirm the robustness of our empirical results and reinforce the validity of the study’s central hypothesis.

**Table 4:** Results of robustness checks.

Independent Variable	Model 3		Model 4	
	Alternative measurement of information enterprise development		Using factor market development as independent variable	
	Coeff. (S.E.)	H.R.	Coeff. (S.E.)	H.R.
Internal goal consensus on innovation	0.037^***^(0.010)	1.038	0.032^***^(0.009)	1.032
Government fiscal resources	0.579(0.420)	1.785	0.469(0.383)	1.598
Regional financial resources	0.008(0.006)	1.008	0.003(0.005)	1.003
Internal government coordination	0.016^***^(0.005)	1.016	0.013^**^(0.005)	1.013
Information enterprise development	−4.998^**^(2.430)	0.007	--	--
Factor market development	--	--	−0.170^***^(0.051)	0.843
Government-business relationship quality	0.266^**^(0.111)	1.304	0.280^**^(0.109)	1.323
Leadership performance demand	0.101^*^(0.053)	1.106	0.098^*^(0.053)	1.103
Corporate social responsibility	0.059^**^(0.029)	1.061	0.060^**^(0.028)	1.062
Market technology	−0.823^***^(0.160)	1.014	−0.760^***^(0.162)	0.468
External knowledge	0.014^*^(0.008)	0.863	0.013(0.008)	1.013
Project level	−0.148(0.467)	1.061	−0.209(0.476)	0.811
Demonstration status	0.413^***^(0.086)	1.511	0.404^***^(0.086)	1.498
Investment size	0.013^***^(0.005)	1.014	0.012^**^(0.005)	1.012
Return mechanism	0.639^***^(0.157)	1.895	0.675^***^(0.158)	1.963
Cooperation duration	−0.376^*^(0.208)	0.687	−0.275(0.209)	0.759
Year FE	Yes		Yes	
*Observations*	736		736	
pseudo *R*^2^	0.077		0.082	

Notes: Robust standard errors are reported in parentheses; ^*,^^**,^^***^ denote significance levels of 10%, 5% and 1%. All models pass the PH risk assumption.

## 5. Conclusions and policy implications

### 5.1. Conclusions

The PPP model has increasingly become a substitute for, or complement to, traditional construction approaches in the realm of digital society development. However, PPP projects in this sector are currently facing significant landing challenges. In the Chinese context, PPP projects are typically initiated by the government and managed through administrative directives issued by national or local authorities. As such, the speed of PPP project landing essentially reflects the effectiveness of policy execution. The policy execution system in which a project is embedded is theoretically a critical factor influencing its progress. Drawing on the theoretical framework proposed by Van Meter and Van Horn, we identify the key components of the policy execution system in digital society PPP projects and constructs a causal mechanism linking this system to project landing speed. Using data from 300 projects, the study employs continuous-time event history analysis to empirically validate the proposed mechanism. The results yield the following findings. (1) Compared with organizational and human factors, material resources such as government fiscal resources and regional financial resources have a relatively limited effect on project landing speed. (2) Among various organizational and actor factors, internal goal consensus on innovation, internal government coordination, government-business relationship quality, leadership performance demand, and corporate social responsibility all exhibit a significant positive correlation with landing speed. (3) However, due to low project profitability and obstacles to mobilizing private sector capital, information enterprise development shows a significant negative correlation with project landing speed.

This research not only identifies the key components of the policy implementation system in digital society PPP projects based on the Van Meter-Van Horn model and establishes their causal relationship with landing speed, but also makes broader theoretical contributions to the field of policy implementation. The study finds that in governance contexts where material resources are relatively abundant, inter-organizational relationships and institutional embeddedness—such as government coordination, government–enterprise relations, and leadership incentives—have a more pronounced impact on execution performance than material inputs. This suggests a need to move beyond the traditional overemphasis on resource provision in policy implementation research and instead focus more on institutional coordination and actor motivation. The study highlights the role of corporate social responsibility as an informal institutional factor in policy execution, offering new theoretical insights into understanding enterprise behavior within execution systems. Finally, the application of PPPs in digital society development—as an emerging policy tool—reflects the growing complexity and technical nature of policy goals. We provide an analytical framework for understanding how such complex goals can be achieved through coordination mechanisms, offering theoretical relevance for the implementation of complex policies in other domains.

### 5.2. Policy implications

The above findings offer the following implications for promoting the successful landing of PPP projects in digital society development.

On one hand, there is a need to move away from traditional resource-intensive development models and focus on cultivating an enabling organizational environment. While resource investment has the potential to accelerate project landing, realizing this potential depends heavily on the development of organizational and human factors. At this stage, local governments should prioritize internal capacity building and the integration of relevant stakeholders, with the aim of constructing a more supportive policy execution system that enables timely, appropriate, and efficient resource allocation. Specifically, local governments should reinforce the principle of “innovation-driven development“ as a key criterion for evaluating the performance and political credibility of subordinate governments and officials. Institutional arrangements should promote stable coordination mechanisms among functional departments to improve administrative efficiency. Cross-sector collaboration among government, enterprises, and academic institutions should be strengthened to form enduring partnerships. Furthermore, governments should advocate for and foster a culture of corporate social responsibility within society and the market.

On the other hand, policy instruments should be leveraged to enhance the intangible (soft) returns for enterprises participating in digital society PPP projects. The findings suggest that the tangible (hard) financial returns available to firms are currently limited, and external market forces may hinder the mobilization of necessary capital. In this context, local governments should implement supportive policies to enhance the public image of private enterprises involved in these projects. By improving their reputation and social standing, such soft returns can help compensate for the lack of hard returns, thereby making PPP projects more attractive to private capital.
